# The Global Burden of Type 2 Diabetes Attributable to Tobacco: A Secondary Analysis From the Global Burden of Disease Study 2019

**DOI:** 10.3389/fendo.2022.905367

**Published:** 2022-07-22

**Authors:** Jianjun Bai, Fang Shi, Yudiyang Ma, Donghui Yang, Chuanhua Yu, Jinhong Cao

**Affiliations:** ^1^ Department of Epidemiology and Biostatistics, School of Public Health, Wuhan University, Wuhan, China; ^2^ Global Health Institute, Wuhan University, Wuhan, China

**Keywords:** type 2 diabetes, tobacco, mortality, world regions, spatial autocorrelation, DALYs - disability-adjusted life years

## Abstract

**Objectives:**

Growing epidemiological studies have reported the relationship between tobacco and health loss among patients with type 2 diabetes (T2D). This study aimed to explore the secular trend and spatial distribution of the T2D burden attributable to tobacco on a global scale to better understand regional disparities and judge the gap between current conditions and expectations.

**Methods:**

As a secondary analysis, we extracted data of tobacco-attributable T2D burden from the 2019 Global Burden of Disease Study (GBD). Joinpoint regression was adopted to determine the secular trend of age-standardized rates (ASR), with average annual percentage change (AAPC). Gaussian process regression (GPR) was used to explore the average expected relationship between ASRs and the socio-demographic index (SDI). Spatial autocorrelation was used to indicate if there is clustering of age-standardized DALY rate (ASDR) with Moran’s I value. Multi-scale geographically weighted regression (MGWR) was to investigate the spatial distribution and scales of influencing factors in ASDR attributable to tobacco, with the regression coefficients for each influencing factor among 204 countries.

**Results:**

Tobacco posed a challenge to global T2D health, particularly for the elderly and men from lower SDI regions. For women, mortality attributable to secondhand smoke was higher than smoking. A downward trend in age-standardized mortality rate (ASMR) of T2D attributable to tobacco was observed (AAPCs= -0.24; 95% CI -0.30 to -0.18), while the ASDR increased globally since 1990 (AAPCs= 0.19; 0.11 to 0.27). Oceania, Southern Sub-Saharan Africa, and Southeast Asia had the highest ASMRs and ASDRs, exceeding expectations based on the SDI. Also, “high-high” clusters were mainly observed in South Africa and Southeast Asian countries, which means a high-ASDR country is surrounded by high-ASDR neighborhoods in the above areas. According to MGWR model, smoking prevalence was the most sensitive influencing factor, with regression coefficients from 0.15 to 1.80.

**Conclusion:**

The tobacco-attributable burden of T2D should be considered as an important health issue, especially in low-middle and middle-SDI regions. Meanwhile, secondhand smoke posed a greater risk to women. Regional disparities existed, with hot spots mainly concentrated in South Africa and Southeast Asian countries.

## Introduction

Diabetes mellitus, characterized by elevated blood glucose, have been one of the leading causes of disease burden worldwide. According to the IDF Diabetes Atlas 10th edition, 537 million individuals (20-79 years of age) are suffering from diabetes, 6.7 million deaths were attributed to diabetes in 2021, and by 2045, there would be 783 million people with diabetes. Type 2 diabetes (T2D), caused by insulin resistance and β cell dysfunction, accounts for around 90% of diabetes cases and leads to a heavy burden on individuals and health systems (1). A 15% increased risk of premature death and an approximately 20-year reduction in life expectancy were observed in patients with T2D (2). Since T2D is closely related to behavioral and metabolic risk factors, avoiding known determinants through early behavioral modification is the most cost-efficient strategy to reduce disease burden.

Growing epidemiological studies have reported that tobacco smoking is one of the most important modifiable risk factors for T2D (3). According to the GBD 2019, smoking is the third leading risk factor for T2D burden worldwide, with 9.9% of the T2D burden attributable to smoking (4). The biological mechanism may involve oxidative stress, inflammatory markers, and glucose metabolism irregularities (5, 6). The 2014 Surgeon General’s Report, for the first time, inferred causal association between smoking and T2D, as well as the potential dose-response relationship (7). A meta-analysis of 22 prospective studies (16,383 patients with T2D) revealed a similar association between smoking and T2D, with a pooled RR of 1.38 for T2D in current smokers compared to never smokers (8). A study in Australia found that smoking and diabetes are associated with an increased risk of mortality and micro- and macrovascular complications, which is intensified when combined (9). Besides, secondhand smoking has been a global health problem and more likely to occur indoors at work or home with active smokers. Secondhand smoke is considered to be associated with an increased risk of T2D (RR=1.22) (3). A national study in China also showed the positive relationship between secondhand smoke and T2D risk (10). Besides, the ultimate purpose of glycemic control is to prevent microvascular and macrovascular complications. Studies have shown that tobacco use is positively associated with important diabetic complications, including cardiovascular disease (CVD), neuropathy, nephropathy, and retinopathy (11). Meanwhile, the association between smoking and diabetes and CVD is well established. Smoking and diabetes interacted with each other in relation to increased risk of CVD events (12, 13). Thus, optimal management of tobacco use and control of blood glucose levels are essential to prevent diabetic complications, while also contributing to a reduction in the burden of cardiovascular disease in the whole population (14).

Given the increasing diabetes burden worldwide, the United Nations adopted Sustainable Development Goals (SDGs) target 3.4 and a series of measures to achieve a 30% reduction of premature mortality from non-communicable diseases (NCDs) by 2030 globally, including diabetes (15). The World Health Organization, the American Diabetes Association Guidelines, and the Italian Diabetes Clinical Guidelines all consider smoking as a preventable risk factor for T2D and support smoking cessation as one of the most important steps in preventing diabetes complications (16–18). Many developing countries such as China, Brazil, and South Africa, have also made great efforts on tobacco control, although progress varied substantially (19, 20). In clinical practice, physicians usually adopted smoking cessation as a basic intervention for long-term care of patients with diabetes. Whereas, some studies showed no significant changes in smoking rates among the diabetic population, comparable to the non-diabetic population (9, 21, 22). Hence, to judge the gap between current conditions and SDGs, it is essential to explore the spatial distribution disparities of tobacco-attributable T2D burden and evaluate the secular trends over the recent period on a global scale, especially among countries or regions with different social-economic levels. To our knowledge, there is no study available giving similar trends on a global scale.

In this study, we investigated the burden of T2D attributable to tobacco on a global scale, examined the secular trends by Joinpoint regression analysis, and explored the spatial distribution disparities through geographic analysis.

## Materials and Methods

### Data Sources

The GBD 2019 provided a systematic and comprehensive annual assessment of 369 diseases and injuries, 87 behavioral, environmental, occupational, and metabolic risk factors among 204 countries or territories from 1990 to 2019. The reliability of the GBD data have been confirmed previously (23–25). The diabetic count reported by the GBD 2019 (460 million) was similar to that of the International Diabetes Federation 2019 (463 million) (26). As a secondary analysis of the GBD, the data of T2D burden attributable to tobacco and related influencing factors were extracted from the GBD 2019, including deaths, DALYs, age-standardized mortality rate (ASMR), age-standardized DALY rate (ASDR), socio-demographic index (SDI), age-standardized smoking prevalence (ASSP) and diabetes treatment index (DTI) among 204 countries or territories and 21 GBD regions. Age-standardized rates (ASR) were calculated by the GBD 2019 global standard population to eliminate the impact of age structure and population differences.

The DTI was used to evaluate the access and quality of diabetes care for a given set of interventions or services, varying from 0 to 100 (27). The SDI was considered a good indicator to reflect the health-related socio-economic developments. It is a composite indicator of lag-distributed income per capita, mean education for those aged 15 and older, and total fertility rate under 25, ranging from 0 to 1 (**Supplementary Material**) (23, 28). Based on the SDI, the 204 countries or territories were divided into five quintiles: low (< 0.46), low-middle (0.46-0.60), middle (0.61-0.69), high-middle (0.70-0.81), and high (> 0.81) SDI regions.

Our study was based on the publicly available the GBD database (GHDx). All data were publicly accessible online at (http://ghdx.healthdata.org/gbd-results-tool). Therefore, ethical approval is not applicable to our study.

### Case Definition

The detailed methodology of the GBD 2019 has been described previously (23, 25, 28, 29). Briefly, to estimate all-cause mortality, cause-specific mortality, and YLLs, the GBD studies utilized standardized data identification, extraction, and processing methods to address data incompleteness, discrepancies in coding practices, and inconsistent age group and sex reports (30). In the GBD 2019, overall diabetes mellitus mortality was estimated using deaths directly attributed to diabetes mellitus. T2D deaths were defined by codes E11-E11.1, E11.3-E11.9 based on the Tenth Revision of the International Classification of Diseases. The GBD 2019 used a Bayesian hierarchical Cause of Death Ensemble model (CODEm) platform to build the best-fitted model with appropriate country-level covariates and analyze 20,830 site-years of vital registration data, and 448 site-years of sample-based vital registration data to estimate T2D mortality. The CoDCorrect process was then conducted to ensure that the cause-specific mortality and all-cause mortality estimates were internally consistent. YLLs were calculated by multiplying deaths by the residual life expectancy at the age of death based on the GBD 2019 reference life table (23, 25).

Nonfatal estimates were generated using data from the systematic literature search, hospital discharge, claims systems, household surveys, cohort studies, and disease registries (23, 30). T2D was defined as “fasting plasma glucose (FPG) ≥ 126 mg/dL (7 mmol/L) or reporting to be on drug or insulin treatment for type 2 diabetes” in the GBD 2019, and the corresponding sequelae were described as well (generic uncomplicated disease, diabetic neuropathy, vision impairment, etc.). Meanwhile, to ensure comparability of data across data sources, the GBD 2019 used MR-BRT analysis for bias adjustment methods to allow a more direct comparison between alternative case definitions (like HbA1c, OGTT, claims data) and/or study designs (23). A compartmental meta-regression tool, DisMod-MR2.1, was then used to synthesize all available data sources to produce internally consistent prevalence estimates. The YLD for each sequela was obtained by multiplying the prevalence and sequela-specific disability weight. After comorbidity correction, the sum of the YLDs for each general sequel denoted the total YLDs for T2D. DALYs were the sum of YLLs and YLDs, referring to all healthy life years lost from onset to death. The GBD generated 95% uncertainty intervals (UI) for all reported data based on the 25th and 975th ordered values of 1,000 draws of the posterior distribution.

Meanwhile, according to the GBD 2019, the burden of T2D attributable to tobacco was divided into two parts: smoking and secondhand smoke. Smoking case definitions were former and current smoking of any tobacco product daily or occasionally. Secondhand smoke was defined as current exposure to secondhand smoke at home, work, or other public places (28). Based on the GBD comparative risk assessment framework, population attributable fractions (PAFs) were used to quantify what proportion of disease burden in a specific population would be reduced if the exposure of certain causal factors were reduced to the theoretical minimum risk exposure level (TMREL) (28, 31). PAFs of disease outcomes were estimated based on exposure data, relative risk of outcomes, and the TMREL. Population surveys were the primary source of exposure data on smoking and secondhand smoke (28). Relative risks were derived from meta-analyses of cohort and case-control studies. The TMRELs for smoking and secondhand smoke were defined as zero. T2D burden attributable to tobacco was calculated by multiplying relevant PAFs by the T2D overall burden for each age-sex-location-year (31).

### Statistical Analysis

We adopted the Joinpoint regression model to determine the secular trend of age-standardized rates. The Joinpoint regression model mainly uses the Grid search method to analyze and establish all possible change points and selects the points with small mean squared errors (MSE) as the joinpoints, which divided the overall trend into several segments (32). The annual percentage change (APC) for each segment, the average annual percentage change (AAPC) for overall trend, and 95% confidence intervals (CIs) were estimated by the Joinpoint model:


$$In\left(ASR\right)=\alpha +\beta_{i}x+\epsilon$$

$$APC=\left(e^{\beta_{i}}-1\right)\times 100$$

$$AAPC=\left(\text{exp}\frac{\sum w_{i}\beta_{i}}{\sum w_{i}}-1\right)\times 100$$

Where *x* represents the calendar year, *β_i_* represent the segmental regression coefficients, and *w_i_* is the interval span of the different segments.

The average relationship between ASRs and SDI was calculated using Gaussian process regression (GPR) model. Gaussian processes are the basic principle behind GPR. Instead of inferring a distribution over the parameters of a parametric function, Gaussian processes could infer a distribution over functions directly, which defines a prior over functions. After having observed some function values, it can be converted into a posterior over functions. The general form is as follow:


p(f|X)=N(f|μ,K)

where *f*=(*f*(*x*_1_),…, (*f*(*xn*)), *μ=*(*m*(*x*_1_),…,*m*(*x*_n_)), *K* is the kernel function. m is the mean function and it is common to use *m*(*x*)=0. Thus, kernel function is the important part of GPR. We chose the classical “Radial Basis kernel function” to conduct the GPR with 10-fold cross validation by the “Kernlab” package of R software. Observed values are the actual disease burden rates in each location-year, while expected values were determined by GPR on the range of rates observed for each level of the SDI (30). We used these estimates of expected ASRs that were predicted based on the full range of the SDI to determine whether observed health patterns deviated from trends associated with changes along the socio-economic development spectrum (33). The associations between expected ASRs and the SDI were explored using the Pearson correlation analysis (30, 34, 35).

In addition, we adopted spatial autocorrelation to explore the spatial distribution of ASDR. Global spatial autocorrelation is used to determine whether the ASDR has aggregation characteristics in overall space, with the Global Moran’s I, ranging from -1 to 1. Global Moran’s I > 0 indicates similar values cluster together in a map; I < 0 indicates dissimilar values cluster together in a map; I = 0 indicates no spatial correlation (36). The calculation formula is as follows:


$$Global\;Moran's\;I=\frac{k\sum_{i=1}^{k}\sum_{j=1}^{k}w_{ij}\left(x_{i}-\bar{x}\right)\left(x_{j}-\bar{x}\right)}{\left(\sum_{i=1}^{k}\sum_{j=1}^{k}w_{ij}\right)\sum_{i=1}^{k}{\left(x_{i}-\bar{x}\right)}^{2}}$$

where *i ≠ j, k* is the number of spatial units involved in the analysis; *x_i_* and *x_j_* represent the observation values of a certain factor *x* in spatial units *i* and *j*, respectively; $\bar{x}$ represents the average value of the attribute value, and *w_ij_* is the spatial weight matrix, calculated by the queen contiguity weight matrix in GeoDa software.

Also, local spatial autocorrelation can locate the extent of spatial hot spots using the LISA cluster map. In LISA cluster map, “high” represents the ASDR being higher than the global average level, “low” represents the ASDR being lower than the average level. “High-high” cluster means a high-ASDR country is surrounded by high-ASDR neighborhoods, while “low-low” cluster means a low-ASDR country is surrounded by low-ASDR neighborhoods.

Furthermore, different processes can operate at different spatial scales and the impact of tobacco on T2D burden may be determined not only by global socio-economic development but also by local specific conditions, such as smoking rates and T2D treatment levels (37). We adopted multi-scale geographically weighted regression (MGWR) to explore the spatial distribution and scales of influencing factors in ASDRs among 204 countries in 2019. The traditional GWR model applies a constant bandwidth to illustrate the impact scales of different factors, which ignores the diversity of impact scales and does not align with the facts (38). MGWR is an extension of the GWR model that allows for exploring the associations at varying spatial scales and achieves that by using a varying bandwidth rather than a single, and constant bandwidth for the entire area, so as to provide more credible estimation results and the diverse impact scales of each factor (39). The calculation formula is as follows:


$$yi=\sum_{j=1}^{k}\beta_{bwj}\left(u_{i},v_{i}\right)x_{ij}+\epsilon_{i}$$

where (*u_i_*,*v_i_*) is the spatial location of the i-th country, *yi* is the response variable, *x_ij_* is the j-th explanatory variable, *β_bwj_* (*u_i_*,*v_i_*) is the j-th coefficient, and *bwj* in *β_bwj_* is the bandwidth used by the regression coefficient of the j-th variable.

In practice, MGWR is usually regarded as a generalized additive model (below), thus it is possible to calibrate the model using the back-fitting algorithm with the classical GWR being the initial estimator (37, 40).


$$y=\sum_{j=1}^{k}f_{j}+\epsilon \;\left(f_{j}=\beta_{bwj}x_{j}\right)$$

where *f_j_* is a smoothing function applied to the j-th explanatory variable. Spatial kernel and bandwidth selection criteria for MGWP were following Adaptive Bisquare and AICc principles. After collinearity analysis, we took SDI, ASSP, and DTI into account for the final MGWR model. The selected variables have relatively low multi-collinearity (all VIFs < 2.0).

Joinpoint regression model was conducted by Joinpoint program (version 4.8.0.1); Pearson correlation and Gaussian Process Regression were performed using R software (version 4.0.2); GeoDa and MGWP were for geographic analysis; ArcGIS for mapping. Detailed information on the above methods can be found in the **Supplementary Materials**. Two-sided P < 0.05 was considered to be statistically significant.

## Results

### Global T2D Burden Attributable to Tobacco Since 1990

In 2019, T2D caused 1.47 (95% UI 1.37 to 1.57) million deaths and 66.30 (55.48 to 79.01) million DALYs worldwide, accounting for 94.96% and 93.54% of those in diabetes mellitus. An estimated 235.43 (163.51 to 299.27) thousand deaths and 11.86 (7.93 to 16.01) million DALYs of T2D were contributed to tobacco.

**Figure 1:**
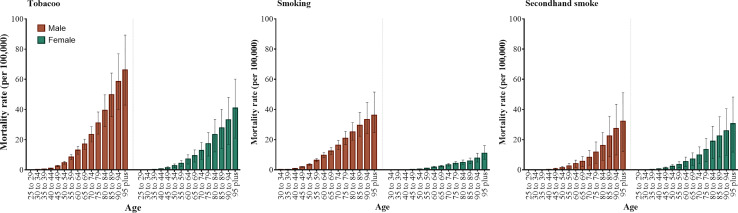
Mortality of T2D attributable to tobacco, smoking, and secondhand smoke by age group and gender. Error bars represent the 95% UIs of mortality.

As shown in **Figure 1**, the mortality of T2D attributable to tobacco increased with age in both genders, and that of men was higher than women. The mortality trends were similar in smoking and secondhand smoke for men. For women, the mortality attributable to secondhand smoke was significantly higher than smoking.

**Table 1** showed ASMR, ASDR, and AAPC of T2D attributed to tobacco from 1990 to 2019 by region. The gender disparities still existed, and the ASMR and ASDR (per 100,000) attributable to tobacco in men were higher than women (3.82 VS 2.14; 183.13 VS 104.47). We observed a declining trend of ASMR attributable to tobacco (AAPCs= -0.24; 95% CI -0.30 to -0.18), while the ASDR remained an increasing trend since 1990 globally (AAPCs= 0.19; 0.11 to 0.27). The ASMR and ASDR of T2D attributable to tobacco were 2.91 (2.01 to 3.70) and 142.00 (94.81 to 191.85) per 100,000 globally in 2019, respectively.

**Table 1:** ASMR, ASDR, and AAPC in type 2 diabetes attributed to tobacco from 1990 to 2019 by regions.

Location	ASMR per 100,000 (95% UI)			ASDR per 100,000 (95% UI)		
	1990	2019	AAPC (95% CI)	1990	2019	AAPC (95% CI)1990-2019
**Global**	3.10 (2.25 to 3.83)	2.91 (2.01 to 3.70)	-0.24* (-0.30 to -0.18)	132.69 (93.52 to 172.97)	142.00 (94.81 to 191.85)	0.19* (0.11 to 0.27)
**Sex**						
Male	3.96 (3.20 to 4.74)	3.82 (2.98 to 4.62)	-0.14* (-0.20 to -0.07)	167.52 (129.34 to 209.19)	183.13 (135.76 to 236.02)	0.25* (0.16 to 0.34)
Female	2.45 (1.47 to 3.29)	2.14 (1.12 to 3.03)	-0.48* (-0.55 to -0.42)	102.44 (58.55 to 143.68)	104.47 (54.42 to 153.68)	0.03 (-0.09 to 0.15)
**SDI regions**						
Low	3.72 (2.42 to 4.91)	3.66 (2.36 to 4.90)	-0.07* (-0.15 to 0.00)	123.83 (81.45 to 164.99)	135.63 (85.21 to 184.83)	0.27* (0.21 to 0.34)
Low-middle	4.28 (2.93 to 5.53)	4.47 (2.96 to 5.86)	0.19 (-0.04 to 0.42)	150.82 (103.56 to 197.01)	173.32 (110.43 to 237.06)	0.42* (0.30 to 0.54)
Middle	3.98 (2.70 to 5.05)	3.97 (2.64 to 5.11)	0.04 (-0.06 to 0.14)	156.36 (105.05 to 206.67)	168.40 (111.25 to 226.88)	0.22* (0.11 to 0.33)
High-middle	2.67 (1.93 to 3.27)	2.15 (1.50 to 2.72)	-0.74* (-0.83 to -0.66)	123.55 (86.86 to 163.25)	124.06 (84.42 to 169.33)	-0.07 (-0.22 to 0.08)
High	2.18 (1.72 to 2.65)	1.31 (1.02 to 1.62)	-1.81* (-1.94 to -1.68)	112.62 (83.24 to 145.62)	105.13 (73.18 to 142.87)	-0.22* (-0.34 to -0.10)
**GBD regions**						
Andean Latin America	2.11 (1.36 to 2.83)	1.96 (1.19 to 2.77)	-0.17 (-0.45 to 0.12)	77.89 (50.89 to 105.84)	80.1 (49.93 to 112.93)	0.08 (-0.04 to 0.20)
Australasia	1.71 (1.34 to 2.08)	0.85 (0.60 to 1.09)	-2.47* (-2.98 to -1.96)	71.42 (53.33 to 92.12)	58.55 (39.05 to 81.77)	-0.66* (-0.76 to -0.57)
Caribbean	5.67 (4.00 to 7.18)	4.07 (2.79 to 5.56)	-1.22* (-1.52 to -0.91)	230.82 (161.40 to 303.76)	201.24 (135.24 to 274.59)	-0.45* (-0.65 to -0.24)
Central Asia	1.66 (1.12 to 2.10)	3.85 (2.67 to 4.97)	2.96* (2.61 to 3.32)	90.05 (59.50 to 122.23)	179.57 (120.4 to 241.27)	2.44* (2.19 to 2.70)
Central Europe	2.57 (1.97 to 3.11)	2.21 (1.60 to 2.86)	-0.54* (-0.69 to -0.39)	147.20 (106.17 to 193.7)	163.66 (112.37 to 225.12)	0.32* (0.25 to 0.40)
Central Latin America	8.06 (5.49 to 10.22)	5.34 (3.39 to 7.26)	-1.42* (-1.72 to -1.12)	306.36 (207.39 to 402.06)	225.79 (141.05 to 315.8)	-1.03* (-1.25 to -0.81)
Central Sub-Saharan Africa	3.89 (2.59 to 5.19)	3.03 (1.96 to 4.25)	-0.90* (-1.00 to -0.80)	128.07 (88.24 to 171.39)	117.00 (76.36 to 162.99)	-0.32* (-0.39 to -0.24)
East Asia	2.12 (1.44 to 2.74)	2.06 (1.46 to 2.67)	-0.03 (-0.35 to 0.28)	112.39 (76.35 to 151.87)	115.87 (79.07 to 158.04)	-0.11 (-0.62 to 0.40)
Eastern Europe	0.68 (0.46 to 0.87)	0.94 (0.66 to 1.21)	1.61* (0.67 to 2.55)	55.67 (37.66 to 77.51)	75.04 (51.75 to 103.78)	1.11* (0.51 to 1.72)
Eastern Sub-Saharan Africa	4.34 (2.95 to 5.68)	3.43 (2.33 to 4.69)	-0.84* (-0.91 to -0.77)	126.44 (85.23 to 167.16)	108.83 (72.58 to 148.09)	-0.54* (-0.59 to -0.49)
High-income Asia Pacific	1.62 (1.27 to 1.94)	0.65 (0.51 to 0.80)	-3.17* (-3.44 to -2.90)	94.86 (69.93 to 123.77)	70.99 (48.23 to 99.14)	-1.10* (-1.28 to -0.91)
High-income North America	2.67 (2.06 to 3.32)	1.80 (1.40 to 2.24)	-1.41* (-1.61 to -1.21)	145.15 (105.76 to 190.91)	130.13 (92.51 to 173.29)	-0.48* (-0.68 to -0.27)
North Africa and Middle East	5.06 (3.37 to 6.58)	4.37 (2.93 to 5.72)	-0.52* (-0.64 to -0.40)	176.65 (119.64 to 232.91)	203.48 (132.64 to 278.18)	0.53* (0.43 to 0.63)
Oceania	19.85 (13.09 to 27.34)	24.86 (15.94 to 34.86)	0.72* (0.61 to 0.83)	629.55 (424.26 to 839.13)	823.21 (545.77 to 1124.19)	0.88* (0.79 to 0.97)
South Asia	4.06 (2.58 to 5.47)	4.24 (2.60 to 5.73)	0.16 (-0.29 to 0.61)	139.01 (91.33 to 186.89)	164.77 (99.68 to 229.89)	0.50* (0.34 to 0.66)
Southeast Asia	7.13 (4.89 to 9.13)	7.49 (4.96 to 9.67)	0.15* (0.04 to 0.26)	229.12 (158.33 to 294.82)	263.72 (177.49 to 346.34)	0.47* (0.34 to 0.59)
Southern Latin America	4.10 (2.89 to 5.14)	3.07 (2.09 to 3.93)	-1.02* (-1.18 to -0.86)	144.12 (102.76 to 186.19)	148.36 (101.28 to 202.52)	0.11* (0.01 to 0.20)
Southern Sub-Saharan Africa	9.04 (6.35 to 11.65)	9.72 (6.16 to 13.03)	0.39 (-0.18 to 0.96)	276.14 (194.84 to 352.02)	290.68 (186.92 to 386.16)	0.29 (-0.15 to 0.74)
Tropical Latin America	7.15 (5.32 to 8.75)	3.85 (2.64 to 5.05)	-2.15* (-2.30 to -2.00)	270.15 (200.07 to 341.72)	156.24 (105.88 to 210.38)	-1.94* (-2.19 to -1.68)
Western Europe	2.20 (1.73 to 2.67)	1.10 (0.84 to 1.36)	-2.42* (-2.50 to -2.33)	101.04 (74.35 to 131.73)	95.45 (64.31 to 133.5)	-0.22* (-0.26 to -0.17)
Western Sub-Saharan Africa	2.62 (1.53 to 3.69)	2.81 (1.66 to 3.88)	0.27* (0.19 to 0.34)	78.52 (46.56 to 109.44)	88.58 (53.35 to 125.72)	0.43* (0.37 to 0.48)

ASMR, age-standardized mortality rate; ASDR, age-standardized DALY rate; SDI, socio-demographic index; AAPC, the average annual percentage change; UI, uncertainty interval; CI, confidence interval.

* means that AAPCs (95% CIs) with "*" represent significance at P < 0.05.

### T2D Burden Attributable to Tobacco Since 1990 by SDI Regions

The ASRs of T2D attributable to tobacco remained the highest among low-middle and middle SDI regions over the study period. In 2019, the highest ASMR and ASDR (per 100,000) were observed in the low-middle SDI region, 4.47 (2.96 to 5.86) and 173.32 (110.43 to 237.06), respectively. The ASRs in the high SDI region remained the lowest. In 2019, the lowest ASMR and ASDR (per 100,000) were 1.31 (1.02 to 1.62) and 105.13 (73.18 to 142.87), respectively (**Table 1**).

**Figure 2:**
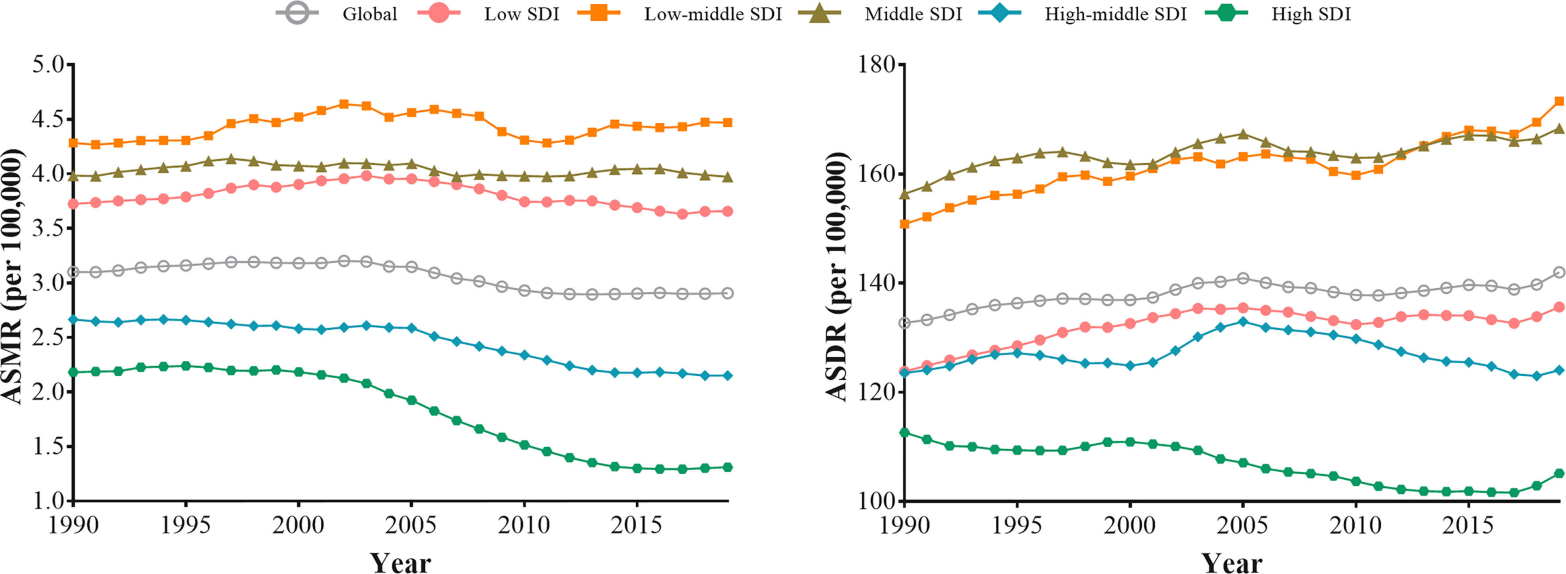
The trends in the ASMR and ASDR for type 2 diabetes attributable to tobacco globally and among five SDI regions from 1990 to 2019.

Between 1990 and 2019, except for low-middle and middle SDI regions, the declining trend of ASMR attributable to tobacco were observed across the other SDI regions, especially after 2015 (**Figure 2**). High SDI regions showed the greatest decline in ASMR attributable to tobacco (AAPC= -1.81; -1.94 to -1.68). While in recent years, the decline in ASMR has stagnated among five SDI regions. Since 1990, low, low-middle, and middle SDI regions have shown rising trends in ASDR attributable to tobacco. Nevertheless, the declining trend was observed in the high SDI region from 1990 to 2019, with AAPC of -0.22 (-0.34 to -0.10). Also, all five SDI regions showed increasing trends of ASDR after 2017 (**Figure 2**). For smoking, similar trends were observed in ASRs. For secondhand smoke, all five SDI regions exhibited a consistent increase in ASDR, particularly in the low-middle SDI regions (**Supplementary Figure 1**).

### T2D Burden Attributable to Tobacco Since 1990 by GBD Regions

Geographic differences existed among 21 GBD regions. The highest ASMR and ASDR (per 100,000) attributable to tobacco occurred in Oceania (24.86 and 823.21), followed by Southern Sub-Saharan Africa (9.72 and 290.68) and Southeast Asia (7.49 and 263.72). Besides, Australasia, high-income Asia Pacific and Eastern Europe were regions with the lowest ASMR (0.85, 0.65 and 0.94) and ASDR (58.55, 70.99 and 75.04). Central Asia showed the most noticeable growth in ASMR and ASDR over the past decades, with AAPCs of 2.96 (2.61 to 3.32) and 2.44 (2.19 to 2.70), respectively. The most significant decrease in ASMR was observed in high-income Asia Pacific (AAPCs= -3.17; -3.44 to -2.90), the most significant reduction in ASDR was in tropical Latin America (AAPCs= -1.94; -2.19 to -1.68), followed by high-income Asia Pacific. The three-segment trends of ASRs among 21 GBD regions are shown in **Supplementary Table 1**.

**Figure 3:**
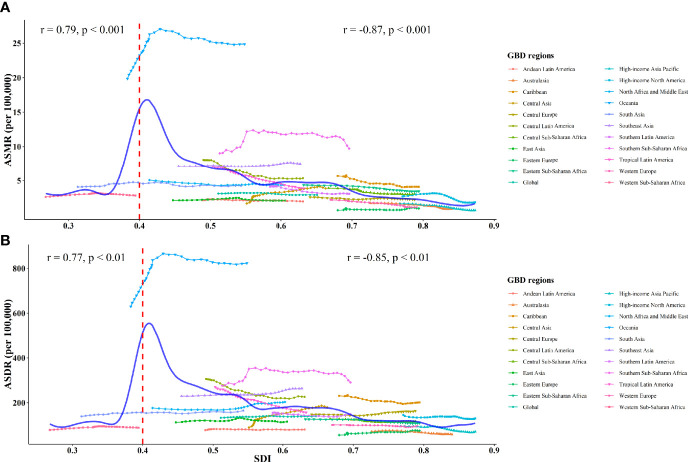
ASMR **(A)** and ASDR **(B)** of type 2 diabetes attributed to tobacco globally and among 21 GBD regions from 1990 to 2019. The solid blue line represents the expected value based on the SDI. The Pearson correlation coefficients and P-values are displayed.

The estimated relationship between the SDI and the expected ASRs of T2D attributable to tobacco was highly positive when the SDI was < 0.40 and highly negative when the SDI was > 0.40 (**Figure 3**). The expected ASRs based on GPR are shown as the blue line in **Figure 3**. The regions above the blue line for ASRs represent a lag behind expected improvements in T2D burden attributable to tobacco. Over the study period, the regions with much higher-than-expected ASRs included Oceania, Southern Sub-Saharan Africa, Southeast Asia, and the Caribbean. While among most other regions, improvements in T2D burden outpaced what would have been expected based on SDI improvements alone, especially in East Asia, Eastern Europe, Andean Latin America, and Australasia.

### Spatial Autocorrelation and MGWR by Countries

Spatial autocorrelation and MGWR were conducted to explore the spatial distribution patterns and influencing factors of the T2D burden attributable to tobacco. Global Moran’s I values demonstrated that positive autocorrelations of tobacco-attributable T2D ASDRs existed (all P < 0.05; **Supplementary Table 2**). Meanwhile, according to local Moran’s I index and LISA clustering map, “high-high” clusters were mainly observed in South Africa, the Kingdom of Saudi Arabia, and Southeast Asian countries, which were the main lower SDI countries (**Supplementary Figure 2**).

The R^2^ (0.846) and Adj-R^2^ (0.819) of MGWR was higher, and the AICc value was lower compared with GWR model (**Supplementary Table 3**), indicating the MGWR result was more reliable. MGWR results suggested that the influencing scale of different factors varied greatly. ASDRs attributable to tobacco were sensitive to the ASSP factor and spatial heterogeneity existed. The influencing scale of ASSP was 43, accounting for 21.08% of the 204 countries or territories, which was close to the sub-continent scale. Once beyond this scale, the coefficient would change. The influencing scale of the SDI was 195, a global scale, suggesting the influence of the SDI on space is relatively stable.

**Figure 4:**
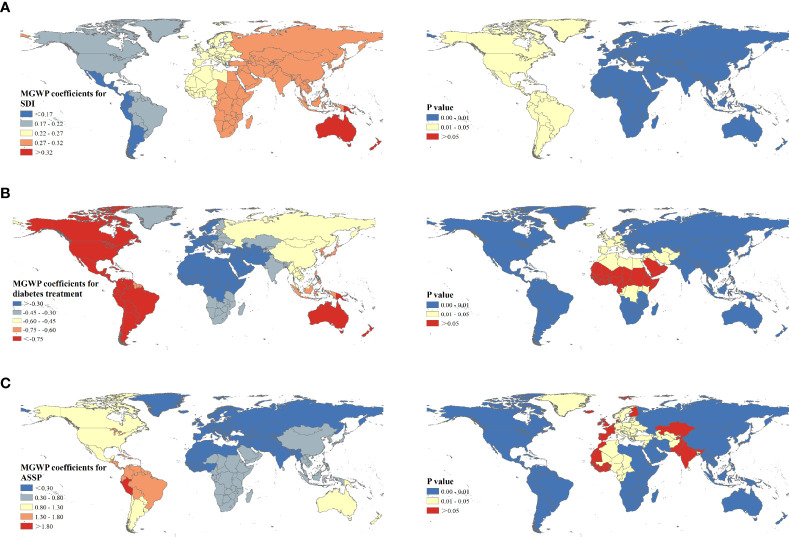
Spatial patterns of regression coefficients and corresponding P values for socio-demographic index **(A)**, diabetes treatment index **(B)**, and age-standardized smoking prevalence **(C)** among 204 countries or territories.

**Figure 4** shows the mapping of coefficients of MGWR and corresponding P-value for the selected factors. The SDI had a positive effect on ASDR of T2D, but the effect was relatively weak, with coefficients from 0.17 to 0.33. Furthermore, a clear directional feature of regression coefficients of the SDI was observed extending in the west-east direction. Also, negative regressions were observed between DTI and ASDRs, ranging from -0.87 to -0.13. DTI was a decisive factor in explaining the ASDR of T2D across North America, South America, and Australia. Additionally, positive regression coefficients were found for ASSP, varying from 0.15 to 1.80. Higher correlations were concentrated in countries from South America (Brazil, Ecuador, and Columbia). Meanwhile, the highest absolute value of coefficient suggested ASSP was the main influencing factor.

## Discussion

In the global analysis of the T2D burden attributable to tobacco, we found a declining trend in the global ASMR attributable to tobacco, whereas the ASDR remained an increasing trend since 1990 globally. The tobacco-attributable T2D burden posed great challenges to the elderly and men from low-middle and middle SDI regions. While secondhand smoke posed a greater risk for women. Meanwhile, “high-high” clusters were mainly observed in South Africa, the Kingdom of Saudi Arabia, and Southeast Asian countries.

Gender disparities were observed and the burden of T2D attributed to tobacco is more remarkable for men, partly because the smoking prevalence in men is higher and men are more likely to be exposed to secondhand smoke (41). Additionally, insulin resistance is more likely to occur in men (42), while estrogen can affect enzyme (CYP2A6) activity and promote nicotine metabolism for women (43). Meanwhile, smoking can also raise serum levels of heavy metals like lead, arsenic, and cadmium, which may affect glucose homeostasis and increase the risk of T2D (6, 44). Secondhand smoke, such as sidestream smoke, is mostly unfiltered and more likely to occur indoors at work or home, and its toxicity increases as it remains in the air for minutes or hours (45). Previous prospective studies have suggested the association between secondhand smoke and T2D risk (46, 47). Also, compared to men, the T2D burden attributable to secondhand smoke in women was higher and secondhand smoke was the main contributor to womens’ tobacco-attributable T2D burden. Probably because daily time, frequency, and total duration of secondhand smoke exposure were higher in women, although secondhand smoke exposure prevalence was lower in women (46). Moreover, a prospective cohort in the US found a 16% higher rate of T2D among female nonsmokers with secondhand smoke exposure than those without (48).

Geographical differences were also detected at the regional and national level. For the high SDI region, the T2D burden attributable to tobacco remained lowest and showed the most significant downward trend since 1990. Higher SDI usually means more government expenditure on T2D, earlier screening, more access to health services, adequate health infrastructure, and people being more health-conscious (35, 36). North America and the Caribbean (415 billion USD) alone accounted for 42.96% of global diabetes health expenditure (966 billion USD) in 2021 (49). Meanwhile, the negative relationships between the SDI and ASRs confirmed the above view when the SDI was>0.40. Low-middle and middle SDI regions showed the highest burden of T2D attributable to tobacco and continued increasing trends. Countries from these regions are mostly developing countries (China, Brazil, India, and Indonesia), which are the main tobacco producing and consuming countries worldwide (50). Despite declines in smoking prevalence observed worldwide, rapid urbanization, population growth, and aging may offset the potential gains of these decreases and drive increases in tobacco-attributable T2D burden among low-middle and middle SDI countries (41). Also, the tobacco-attributable T2D burden did not seem to follow the expected relationship with the SDI in low SDI regions, which was lower than that in low-middle and middle SDI regions. The absence of universal healthcare coverage, shortage of medical personnel, and inability to timely diagnose may cause missed diagnosis, under-reporting, and underestimates regarding the burden T2D attributable to tobacco use in low SDI regions (34, 51).

Additionally, “high-high” clusters were mainly observed in South Africa, the Kingdom of Saudi Arabia, and Southeast Asian countries; and the tobacco-attributable T2D burden in these regions was much higher than the expected value-based SDI. This may be explained by the relatively high tobacco use rate and poor T2D treatment. The Southeast Asia region has 26% of the world’s population and was one of the largest tobacco producers and consumers, with 250 million tobacco smokers and a massive number of smokeless tobacco users while at the same time, this region accounted for just 1% of the global diabetes health expenditure (49, 52). Meanwhile, the tobacco use rate among adolescents (13-15 years old) remains high in Southeast Asia, with the highest rate being 30.3% in Bhutan (53). The epidemiologic study has shown that Asians, especially South Asians, are more genetically susceptible to T2D (54). South Africa has been experiencing rapid socio-economic growth and the transformation of disease burden to NCD in recent years. However, medical and health undertakings seem to lag behind socio-economic development and fail to meet the needs of NCD prevention and control (55). In 2019, 59.7% of people with diabetes were undiagnosed in Africa (26). Also, late diagnosis and poor glycemic control exacerbated the burden of diabetes and complications in South Africa (56).

Across the 204 countries or territories, smoking prevalence is the most sensitive and direct factor affecting tobacco-attributable T2D burden, followed by diabetes treatment. Over the past decade, many effective tobacco-control initiatives and interventions have been conducted to address the tobacco epidemic, including tobacco taxes, smoking bans in public places, smoking cessation interventions, and the WHO Framework Convention on Tobacco Control (57, 58). The overall smoking prevalence has decreased, but the number of smokers is still increasing due to population growth. Multinational studies showed that the proportion of women smoking was gradually increasing (59). Meanwhile, tobacco control policies may be blocked in developing countries, where tobacco revenues are essential to the national economy (60). In clinical guidelines, smoking cessation is recognized as an essential intervention of the long-term care of patients with diabetes. However, the physician may encounter obstacles in promoting smoking cessation to prevent diabetes (14). A national cohort study in Australia found similar prevalence of smoking among diabetics (13.5%) as general Australian population (13.8%), partially suggesting poor adherence to primary and/or secondary prevention recommendations for smoking cessation among the diabetic population (9). In the NHANES study of 24,649 participants, the age-adjusted smoking rate was 25.7% among diabetics and 24.1% among non-diabetics (21). In Africa, the prevalence of smoking among diabetics was 12.9%, also similar to the prevalence of smoking in the general African population (12%) as reported by the WHO during the same period (61). This may be partly because patient concerns about weight gain and withdrawal effects after cessation, and some studies have shown that short-term weight gain after quitting smoking may increase the risk of T2D (62, 63). Additionally, smokers may have insufficient understanding of diabetes, and even if they are aware of the harm of the disease, they may continue to smoke. Smoking, especially continued smoking after diagnosis of diabetes, was independently associated with diabetes complications (22, 64). Therefore, physicians should advocate for smoking cessation interventions in the early stages and combine other interventions such as diet, exercise, and reducing the cost of smoking cessation treatment for patients to maximize the benefits of smoking cessation for diabetes improvement. Besides, most interventions focus on high-risk groups, but recent studies have shown that strategies focusing on detecting and treating high-risk groups are not enough (65). Diabetes “prevention” is often just a “delay” for high-risk groups. More measures should focus on population-based primary care strategies, targeting preventable risk factors that are easily modifiable, particularly tobacco use. Meanwhile, primary diagnosis and surveillance of T2D should be strengthened to improve data reliability among low SDI countries.

The GBD studies came up with comprehensive quality estimates of global disease burden and fill a gap where actual data on disease burden are sparse or unavailable, yet several limitations should be acknowledged. Data on tobacco use were obtained through self-reporting, which may lead to underestimation for population groups with low social acceptance of smoking, especially among women in Asia and Africa (66). Additionally, the accuracy and robustness of the GBD estimate largely depend on the quality and quantity of data used in the modeling. Vital registration, verbal autopsy, and statistics systems are critical sources of vital statistics for mortality rates. However, the population coverage with these systems was disappointing among low-income regions, which may lead to underestimating the T2D burden, although the GBD has conducted many adjusted methods to reduce such bias. Since our study is based on the population level, ecological fallacy might emerge and the relationship between mortality, DALYs and the SDI, ASSP, and DTI, although explanatory, cannot be considered as a causality. Finally, as a secondary analysis of the GBD data, we have no additional detailed covariable data to control the bias, such as race, education and occupation.

## Conclusions

Tobacco should be regarded as an essential and preventable risk factor for the burden of T2D, especially in low-middle and middle SDI regions. Great efforts have been made on tobacco control and a declining trend of ASMR of T2D attributable to tobacco was observed, while the ASDR of T2D increased globally. Gender and regional disparities existed. Tobacco-attributable T2D burden posed great challenges to the elderly and men while secondhand smoke posed a greater risk to women. Hot spots were concentrated in South Africa, the Kingdom of Saudi Arabia, and Southeast Asia and needed more attention with supportive policies to lessen the T2D burden. Also, low SDI regions should increase their health investment in NCDs and strengthen the capacity of diabetes diagnosis and surveillance.

## Data Availability Statement

The datasets presented in this study can be found in online repositories. The names of the repository/repositories and accession number(s) can be found in the article/**Supplementary Material**.

## Ethics Statement

Our study was based on a publicly available GBD database (GHDx). No patients, the public or animals were involved in the design, or conduct, or reporting, or dissemination plans of our study. All data were publicly open access online at (http://ghdx.healthdata.org/gbd-results-tool). Therefore, ethical approval is not applicable for our study.

## Author Contributions

Study design: CY and JC. Data collection: JB. Data analyses: JB, and FS. Results visualisation: JB. Results interpretations: All authors. Manuscript writing: JB. Manuscript revising: JB, FS, YM, DY, CY, and JC. All authors contributed to the article and approved the submitted version.

## Funding

The study was funded by the National Natural Science Foundation of China [grant number 82173626, 81773552], Wuhan Total Health Cost Accounting Project 2017-2020 [grant number WHWSZFY2021], Health commission of Hubei Province scientific research project [grant number WJ2019H304].

## Conflict of Interest

The authors declare that the research was conducted in the absence of any commercial or financial relationships that could be construed as a potential conflict of interest.

## Publisher’s Note

All claims expressed in this article are solely those of the authors and do not necessarily represent those of their affiliated organizations, or those of the publisher, the editors and the reviewers. Any product that may be evaluated in this article, or claim that may be made by its manufacturer, is not guaranteed or endorsed by the publisher.

## Abbreviations

T2D, type 2 diabetes; GBD, Global Burden of Diseases; DALYs, disability-adjusted life years; ASMR, age-standardized mortality rate; ASDR, age-standardized DALY rate; SDI, socio-demographic index; ASSP, age-standardized smoking prevalence; DTI, diabetes treatment index; ASR, Age-standardized rate; APC, annual percentage change; AAPC, the average annual percentage change; GPR, Gaussian Process Regression; MGWR, Multi-scale geographically weighted regression.
