# Supplementary material for: The Global Burden of Type 2 Diabetes Attributable to Tobacco: A Secondary Analysis From the Global Burden of Disease Study 2019
Source: Front Endocrinol (Lausanne). 2022 Jul 22;13:905367. doi: 10.3389/fendo.2022.905367 (PMC9355706; doi:10.3389/fendo.2022.905367)
Supplement: Supplementary file 1 [file DataSheet_1.docx]

Supplementary Material

Table of Contents

[Supplementary Figures and Tables 1](#_Toc102136516)

[Supplementary Figures 1](#_Toc102136517)

[Supplementary Tables 3](#_Toc102136518)

[Joinpoint regression models 5](#_Toc102136519)

[Gaussian process regression (GPR) 7](#_Toc102136520)

[Spatial autocorrelation analysis 9](#_Toc102136521)

[Multi-scale geographically weighted regression (MGWR) 11](#_Toc102136522)

[Socio-Demographic Index (SDI) Definitions and Method 13](#_Toc102136523)

[References 19](#_Toc102136524)

# Supplementary Figures and Tables

## Supplementary Figures

**Supplementary Figure 1.** The trends in the ASMR and ASDR for type 2 diabetes attributable to smoking (A) and secondhand smoke (B) globally and among five SDI regions from 1990 to 2019.


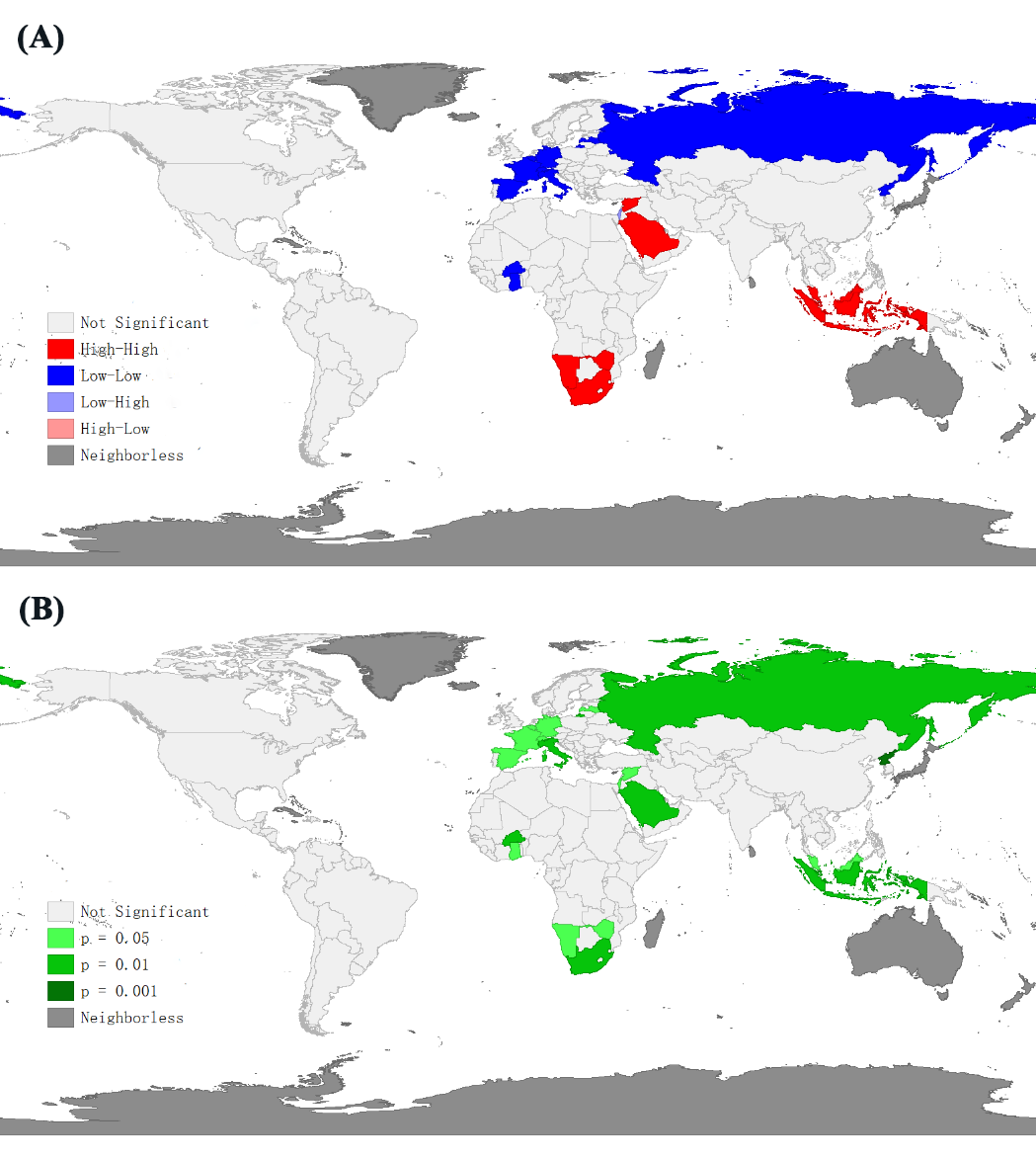


**Supplementary Figure 2.** LISA cluster map (A) and LISA significance map (B) of the ASDR in type 2 diabetes attributable to tobacco in 2019.

## Supplementary Tables

**Supplementary Table 1.** Three-segment trends in the ASMR and ASDR of type 2 diabetes attributable to tobacco among 5 SDI regions and 21 GBD regions from 1990 to 2019.

| **ASMR** | APCs Segment 1 | APCs Segment 2 | APCs Segment 3 |
| --- | --- | --- | --- |
| **Global** | 0.24* (0.18 to 0.3) | -1.28* (-1.44 to -1.12) | 0.03 (-0.10 to 0.17) |
| **SDI regions** |  |  |  |
| Low | 0.52* (0.46 to 0.58) | -0.95* (-1.24 to -0.65) | -0.41* (-0.53 to -0.29) |
| Low-middle | 0.56* (0.44 to 0.69) | -1.88* (-3.41 to -0.34) | 0.46* (0.16 to 0.77) |
| Middle | 0.57* (0.3 to 0.84) | -0.28* (-0.41 to -0.14) | 0.046 (-0.12 to 0.21) |
| High-middle | -0.24* (-0.31 to -0.18) | -1.96* (-2.17 to -1.74) | -0.36* (-0.64 to -0.08) |
| High | -0.19* (-0.35 to -0.04) | -4.24* (-4.41 to -4.07) | 0.26 (-0.34 to 0.88) |
| **GBD regions** |  |  |  |
| Andean Latin America | 1.97* (0.3 to 3.67) | -0.8* (-1.21 to -0.39) | -0.27* (-0.54 to -0.01) |
| Australasia | -1.38* (-1.81 to -0.95) | -4.05* (-4.47 to -3.62) | 0.64 (-3.86 to 5.36) |
| Caribbean | -0.003 (-1.21 to 1.22) | -2.84* (-3.75 to -1.94) | -0.9* (-1.1 to -0.69) |
| Central Asia | 11.08* (9.56 to 12.62) | 2.25* (2.14 to 2.36) | -4.17 (-8.3 to 0.14) |
| Central Europe | -1.38* (-1.55 to -1.21) | 1.38* (0.77 to 2) | -0.72* (-0.89 to -0.55) |
| Central Latin America | -0.1671 (-1.84 to 1.53) | -2.44* (-2.65 to -2.23) | 0.15 (-0.52 to 0.82) |
| Central Sub-Saharan Africa | -0.94* (-1.2 to -0.68) | -1.67* (-1.86 to -1.49) | -0.23* (-0.36 to -0.11) |
| East Asia | 1.22* (1.08 to 1.35) | -4.67* (-7.55 to -1.7) | -0.43* (-0.64 to -0.22) |
| Eastern Europe | 6.93* (1.11 to 13.07) | -1.67* (-2.44 to -0.9) | 4.57* (3.22 to 5.95) |
| Eastern Sub-Saharan Africa | -0.33* (-0.47 to -0.18) | -1.37* (-1.45 to -1.29) | -0.5* (-0.65 to -0.34) |
| High-income Asia Pacific | -2.19* (-2.39 to -1.99) | -5.06* (-5.34 to -4.77) | -0.06 (-2.4 to 2.34) |
| High-income North America | 1.69* (1.42 to 1.97) | -4.61* (-4.83 to -4.39) | 0.35 (-0.55 to 1.26) |
| North Africa and Middle East | -1.48* (-1.64 to -1.32) | 0.51* (0.19 to 0.84) | -0.27* (-0.47 to -0.08) |
| Oceania | 2.72* (2.35 to 3.09) | 1.6* (1.36 to 1.83) | -0.68* (-0.79 to -0.58) |
| South Asia | 1.11* (0.87 to 1.35) | -3.28* (-6.19 to -0.29) | 0.04 (-0.54 to 0.62) |
| Southeast Asia | -0.03 (-0.09 to 0.02) | 0.83* (0.64 to 1.01) | -0.80 (-1.67 to 0.08) |
| Southern Latin America | 0.32* (0.09 to 0.54) | -2.97* (-3.22 to -2.71) | 0.002 (-0.46 to 0.47) |
| Southern Sub-Saharan Africa | 3.92* (3.15 to 4.7) | -0.43* (-0.78 to -0.08) | -5.2* (-9.79 to -0.38) |
| Tropical Latin America | -0.89* (-1.75 to -0.03) | -2.17* (-2.43 to -1.92) | -2.47* (-2.61 to -2.33) |
| Western Europe | -2.08* (-2.16 to -2.00) | -3.45* (-3.57 to -3.32) | -1.01* (-1.39 to -0.63) |
| Western Sub-Saharan Africa | 1.66* (1.5 to 1.81) | 0.88* (0.65 to 1.12) | -0.82* (-0.88 to -0.76) |
| **ASDR** | APCs Segment 1 | APCs Segment 2 | APCs Segment 3 |
| **Global** | 0.35* (0.29 to 0.40) | -0.34* (-0.65 to -0.02) | 0.29* (0.12 to 0.45) |
| **SDI regions** |  |  |  |
| Low | 0.69* (0.62 to 0.75) | -0.29* (-0.50 to -0.08) | 0.11 (-0.01 to 0.23) |
| Low-middle | 0.51* (0.43 to 0.59) | -0.47 (-1.09 to 0.15) | 0.78* (0.59 to 0.97) |
| Middle | 1.07* (0.29 to 1.86) | 0.06 (-0.03 to 0.14) | 0.27 (-0.07 to 0.61) |
| High-middle | 0.08 (-0.05 to 0.22) | 1.37* (0.33 to 2.41) | -0.59* (-0.69 to -0.49) |
| High | -0.09 (-0.21 to 0.03) | -0.7* (-0.84 to -0.54) | 0.61* (0.06 to 1.16) |
| **GBD regions** |  |  |  |
| Andean Latin America | 1.28* (0.78 to 1.79) | -0.41* (-0.57 to -0.26) | 0.07 (-0.07 to 0.22) |
| Australasia | 1.43* (1.06 to 1.81) | -1.65* (-1.74 to -1.57) | -0.16 (-0.35 to 0.03) |
| Caribbean | 0.56 (-1.07 to 2.23) | -0.95* (-1.05 to -0.84) | 0.49* (0.00 to 0.97) |
| Central Asia | 6.56* (5.28 to 7.85) | 2.21* (2.12 to 2.31) | -1.18 (-2.99 to 0.66) |
| Central Europe | -0.32* (-0.41 to -0.24) | 1.38* (1.13 to 1.64) | 0.36* (0.24 to 0.48) |
| Central Latin America | -0.55 (-1.75 to 0.66) | -1.87* (-2.02 to -1.72) | 0.53* (0.04 to 1.02) |
| Central Sub-Saharan Africa | -0.41* (-0.54 to -0.28) | -1.24* (-1.49 to -0.99) | 0.24* (0.16 to 0.33) |
| East Asia | 0.39* (0.06 to 0.73) | 2.63 (-2.33 to 7.85) | -1.02* (-1.23 to -0.80) |
| Eastern Europe | 4.18* (2.34 to 6.05) | -0.93 (-6.28 to 4.73) | 0.85* (0.71 to 0.98) |
| Eastern Sub-Saharan Africa | -0.10 (-0.21 to 0.01) | -0.97* (-1.03 to -0.9) | -0.37* (-0.47 to -0.27) |
| High-income Asia Pacific | -0.15 (-0.43 to 0.14) | -2.39* (-2.69 to -2.1) | -0.48* (-0.94 to -0.02) |
| High-income North America | -2.55* (-4.23 to -0.83) | 0.32* (0.13 to 0.52) | -0.79* (-0.97 to -0.6) |
| North Africa and Middle East | -0.39* (-0.53 to -0.25) | 1.8* (1.56 to 2.03) | 0.41* (0.21 to 0.61) |
| Oceania | 2.49* (2.26 to 2.73) | 1.78* (1.54 to 2.02) | -0.43* (-0.52 to -0.35) |
| South Asia | 1.01* (0.79 to 1.22) | -0.23 (-0.57 to 0.12) | 0.60* (0.29 to 0.92) |
| Southeast Asia | 0.2* (0.15 to 0.25) | 1.73* (1.15 to 2.31) | 0.26 (-0.33 to 0.84) |
| Southern Latin America | 0.73* (0.61 to 0.85) | -1.07* (-1.18 to -0.96) | 1.84* (1.38 to 2.29) |
| Southern Sub-Saharan Africa | 3.38* (2.65 to 4.11) | -0.26* (-0.51 to 0.00) | -4.37* (-8.01 to -0.59) |
| Tropical Latin America | -2.01* (-2.16 to -1.85) | -1.12 (-3.57 to 1.38) | -2.05* (-2.16 to -1.93) |
| Western Europe | -0.19* (-0.23 to -0.15) | -0.71* (-0.78 to -0.64) | 0.79* (0.58 to 1.00) |
| Western Sub-Saharan Africa | 1.65* (1.54 to 1.76) | 0.91* (0.73 to 1.08) | -0.51* (-0.55 to -0.46) |

UR, Uncertainty interval; ASMR, age standardized mortality rate; ASDR, age standardized DALY rate; APC, annual percentage change; SDI, socio-demographic index; *significant at 5% level of significance

**Supplementary Table 2.** Global Moran's I value of ASDR attributable to tobacco from 1990 to 2019.

| Year | Moran's I | Mean | SD | Z Score | P Value | Aggregation |
| --- | --- | --- | --- | --- | --- | --- |
| 1990 | 0.306 | -0.0066 | 0.0608 | 5.1448 | 0.002 | yes |
| 2000 | 0.3577 | -0.0068 | 0.0595 | 6.1256 | 0.001 | yes |
| 2010 | 0.3382 | -0.0080 | 0.0588 | 5.8897 | 0.001 | yes |
| 2019 | 0.3702 | -0.0085 | 0.0594 | 6.3804 | 0.001 | yes |

**Supplementary Table 3.** R^2^, Adj-R^2^, Residual sum of squares and AICc of OLS, GWR and MGWR

| Model | R^2^ | Adj-R^2^ | Residual sum of squares | AICc |
| --- | --- | --- | --- | --- |
| OLS | 0.593 | 0.588 | 100.607 | 489.357 |
| GWR | 0.814 | 0.785 | 45.857 | 364.547 |
| MGWR | 0.846 | 0.819 | 38.133 | 327.996 |

OLS, ordinary least squares; GWR, geographically weighted regression; MGWR, Multi-scale geographically weighted regression.

#

# Joinpoint regression models

Joinpoint regression models are also known as Broken-line, Piecewise regression, etc. It was first proposed by Kim et al in 1998 and allows for analysis of changes in trends in data. The Joinpoint regression model is applied to disease epidemiology studies by using the study period as the independent variable in the regression, creating segmental regressions based on the temporal characteristics of disease manifestations, and fitting trends to the data points in each segment to provide a comprehensive assessment of disease trends over time [1].

1. *Model principles*

Joinpoint regression model requires the dependent variable to be a numerical variable that follows a normal or Possion distribution. There are two main types of models, linear and log-linear. In medical research, the dependent variable can mostly obey the exponential or Poisson distribution, so the log-linear model is more commonly used. The model used in this paper is the log-linear model, with the equation:

$$E\left[ y | x \right]=e^{\beta_{0}+\beta_{1}x+\delta_{1}{(x-\tau_{1})}^{+}+\ldots+\delta_{k}{(x-\tau_{k})}^{+}}$$

where *y* represents the dependent variable, *x* represents the independent variable and (*x, y*) represents a set of data. *β_0_* is the invariant parameter, *β_1_* represents the regression coefficient, *δ_k_* is the regression coefficient of the segmental function. The $\tau_{k}$ are the unknown joinpoints, k is the number of joinpoints. When $x-\tau_{k}$>0, ${(x-\tau_{k})}^{+}$=$x-\tau_{k}$, otherwise ${(x-\tau_{k})}^{+}=0$.

1. *Model construction and optimization methods*

Grid Search Method (CSM) is the default modeling method adopted by Joinpoint. CSM divides the research data into grids, and each grid intersection corresponds to a planning scheme. Then, in the set segment, the performance indicators of the corresponding equations are calculated point by point with a fixed step size to determine the optimal function. In short, Joinpoint model establishes all possible segment joinpoints through CSM method, and calculate the corresponding sum of squares errors (SSE) and mean squared errors (MSE) for each possible segmental function. Select the grid point with the smallest MSE as the joinpoint of the segmental function, and the equation is fitted according to the selected joinpoint and segmental function.

The Monte Carlo permutation test is used by default for model optimization. Before modeling, set the number range of joinpoints (*kmin and kmax*). *kmin* represents the minimum number of joinpoints, which is generally 0. *kmax* indicates the maximum number of joinpoints. In Joinpoint regression model, null hypothesis H0 is: the number of joinpoints k=ka, and alternative hypothesis H1 is: the number of join points k = kb. The permutation test starts from ka=kmin and kb=kmax. If H0 is rejected, k=ka+1 is set to continue the test. If H0 is not rejected, set k= kb-1 and then check until ka= kb. Then the preferred number of joinpoints k=ka=kb is determined. To ensure the stability of the results and consider the computing time, the system default number of permutation test is 4500 times. Because of the multiple tests involved, Bonferroni method was used to adjust for the statistical significance level α=0.05[2].

1. *Indicator calculation*

In Joinpoint regression model, the Annual Percent change (APC) and its 95% Confidence interval (CI) are used to evaluate the trends of different segments. The Average annual percent change (AAPC) and its 95%CI were used to evaluate the average change trend in the whole study period.

For the log-linear $In\left( ASR \right)= \alpha+\beta_{i}x+\varepsilon$ model, APC is calculated by

$$APC=\left( e^{\beta_{i}}-1 \right)\times100$$

The upper and lower limits of 100(1-α) % are:

$${APC}_{L\left( \alpha\right)}=100(e^{\beta_{i}-st_{d}^{-1}\left( 1-\frac{\alpha}{2} \right)}-1)$$

$${APC}_{U\left( \alpha\right)}=100(e^{\beta_{i}+st_{d}^{-1}\left( 1-\frac{\alpha}{2} \right)}-1)$$

where $\beta_{i}$ and *s* represent the regression coefficients and their standard errors, respectively, *d* denotes the degrees of freedom, and *t_d_*(*q*) represents the value of the q-th percentile in the *t* distribution with degree of freedom *d*.

The AAPC and its confidence interval are calculated as

$$AAPC=\left( exp(\frac{{\sum w_{i}\beta}_{i}}{\sum w_{i}})-1 \right)\times100$$

$${AAPC}_{L\left( \alpha\right)}=\exp\left[ \ln\left( \left( \frac{AAPC}{100} \right)+1 \right)-Z_{{1-}_{\frac{\alpha}{2}}}\sqrt{\sum\tilde{w}_{i}^{2}\tilde{\sigma}_{i}^{2}} \right]-1$$

$${AAPC}_{U\left( \alpha\right)}=\exp\left[ \ln\left( \left( \frac{AAPC}{100} \right)+1 \right)＋Z_{{1-}_{\frac{\alpha}{2}}}\sqrt{\sum\tilde{w}_{i}^{2}\tilde{\sigma}_{i}^{2}} \right]-1$$

where $w_{i}$ is the interval span of the different segmental functions, $\beta_{i}$ is the segmental regression coefficient with variance $\tilde{\sigma}_{i}^{2}$, the normal weighting factor $\tilde{w}_{i}=w_{i}/\sum w_{i}$, and *Zα* is the αth percentile value under the normal distribution[3].

# Gaussian process regression (GPR)

A Gaussian process is a collection of random variables, any finite number of which have consistent Gaussian distributions [4].

Gaussian processes (GPs) could be used to infer a distribution over functions directly rather than a distribution over the parameters of a parametric function. A Gaussian process is used to construct a prior over functions, at the core of GP prediction is the covariance function or kernel, capturing the output covariance at different pairs of input points. It could be converted into a posterior over functions after seeing certain function values. GP regression is the inference of continuous function values in this context; GPs could also be adapted for classification [5; 6].

A Gaussian process is a random process where any point x∈ R^d^ is assigned a random variable $f(x)$, and the joint distribution of a finite number of these variables $p(f\left( x_{1} \right),\ldots,f(x_{n}))$ is itself Gaussian:

$$p(f|X)=N(f|\mu,K)$$

In the Equation, $f=(f\left( x_{1} \right),\ldots,f\left( x_{n} \right))$, $\mu=(m\left( x_{1} \right),\ldots,m(x_{n}))$, and $K_{ij}=k(x_{i},x_{j})$. m is the mean function and it is common to use $m\left( x \right)=0$ as GPs are flexible enough to model the mean arbitrarily well. $K$ is a positive definite kernel function or covariance function, capturing the input dependence of the target statistics. Thus, a Gaussian process is a distribution over functions whose shape (smoothness, …) is defined by $K$. If points $x_{i}$and $x_{j}$ are considered similar by the kernel the function values at these points, $f\left( x_{i} \right)$ and $f\left( x_{j} \right)$, can be expected to be similar too.

Given a training dataset with noise-free function values f at inputs X, a GP prior can be converted into a GP posterior $p(f_{*}|X_{*},X,f)$ which can then be used to make predictions $f_{*}$ at new inputs $X_{*}$. By the definition of GP, the joint distribution of observed values f and predictions $f_{*}$ is again a Gaussian which can be partitioned into

$$\binom{f}{f_{*}}\sim N\left( 0,\left( \begin{matrix} K & K_{*} \\ K_{*}^{T} & K_{**} \end{matrix} \right) \right)$$

where $K_{*}=k(X,X_{*})$ and $K_{**}=k(X_{*},X_{*})$. With *N* training data and *N_∗_* new input data K is a N×N matrix, K_∗_ a N×N_∗_ matrix, and K_∗∗_ a N_∗_×N_∗_ matrix. Using the Bayesian rules for conditioning Gaussians, the predictive distribution is given by

$$p\left( f_{*} | X_{*}, X, f \right)=N\left( f_{*} | \mu_{*}, \sum_{*} \right)$$

$$\mu_{*}=K_{*}^{T}K^{-1}f$$

$$\sum_{*}=K_{**}-K_{*}^{T}K^{-1}K_{*}$$

$\mu_{*}=K_{*}^{T}K^{-1}f$ is actually a linear function of the observation point, while $\sum_{*}=K_{**}-K_{*}^{T}K^{-1}K_{*}$ means “Predictive uncertainty = prior uncertainty$-$reduction in uncertainty”, the first part of the covariance term is our a priori covariance, and the subtracted latter term actually represents the reduction in uncertainty of the distribution of the function after seeing the observations.[7; 8]

We used the “kernlab” package of R software to conduct the Gaussian process regression. We the “Radial Basis kernel function” to conduct the GPR

$$k\left( x_{i},x_{j} \right)=\sigma_{f}^{2}exp(-\frac{1}{2l^{2}}\left( x_{i}-x_{j} \right)^{T}(x_{i}-x_{j}))$$

The hyperparameter value would be given automatically according to the algorithm based on “kernlab” package. The 10-fold cross validation on the training data is performed to assess the quality of the model[9].

**The Gaussian process regression model was fitted by the “kernlab” package of R software:**

gausspr (x, y, type= regression, kernel="rbfdot", kpar="automatic", tol=0.001, cross=10, variance.model = FALSE, …)

**Arguments**

**x** a symbolic description of the model to be fit or a matrix or vector when a formula interface is

**y** a response vector with one label for each row/component of x. Can be either a factor (for classification tasks) or a numeric vector (for regression).

**type** "classification" or "regression".

**kernel** the kernel function used in training and predicting. rbfdot Radial Basis kernel function "Gaussian"

**tol** tolerance of termination criterion (default: 0.001)

**cross** a k-fold cross validation on the training data is performed to assess the quality of the model.

**variance.model** build model for variance or standard deviation estimation (only for regression) (default : FALSE)

**Author(s)**

Alexandros Karatzoglou alexandros.karatzoglou@ci.tuwien.ac.at

**References**

C. K. I. Williams and D. Barber

Bayesian classification with Gaussian processes.

IEEE Transactions on Pattern Analysis and Machine Intelligence, 20(12):1342-1351, 1998

http://www.dai.ed.ac.uk/homes/ckiw/postscript/pami_final.ps.gz

# Spatial autocorrelation analysis

Global and local spatial autocorrelation analysis are two types of spatial autocorrelation statistics that is a basic technique for evaluating geographical data. Spatial dependency refers to the degree of connection between data at a certain place and data at other locations. The global index assesses the overall trend of spatial correlation of unit attribute values in geographically nearby places throughout the research region. The calculation formula is as follows:

$${Moran}^{'}I=\frac{k\sum_{i=1}^{k} \sum_{j=1}^{k} w_{ij}(x_{i}-\bar{x})(x_{j}-\bar{x})}{(\sum_{i=1}^{k} \sum_{j=1}^{k} w_{ij})\sum_{i=1}^{k} {(x_{i}-\bar{x})}^{2}}$$

Where *i ≠ j, k* is the number of spatial units involved in the analysis; $x_{i}$ and $x_{j}$ represent the observation values of a certain factor *x* in spatial units *i* and *j*, respectively; $\bar{x}$ represents the average value of the attribute value, and $w_{ij}$ is the spatial weight matrix, calculated by the queen contiguity weight matrix in GeoDa software, indicating the proximity or distance relationship between units *i* and *j*.

The global Moran index value ranges from −1 (for negative spatial auto-correlation) to +1 (for positive spatial auto-correlation). Under a given significance level (generally 0.05), positive spatial autocorrelation is when similar values cluster together in a map, negative spatial autocorrelation is when dissimilar values cluster together in a map. When the event of interest is randomly distributed in the geographic space, the value of *I* is expected to be approximately zero [10].

In addition, the spatial correlation of different units across the entire study area may have its own characteristics. Therefore, the local Moran index was calculated, and the local indicator of spatial auto-correlation (LISA) diagram is used to indicate local spatial associations, which is helpful for spotlighting clusters of meso-regions with significant spatial auto-correlation [10; 11]. The calculation formula of local Moran index is as follows:

$$I_{i}=\frac{x_{i}-\bar{x}}{S^{2}}\sum_{j=1}^{k} w_{ij}(x_{j}-\bar{x})$$

$$S^{2}=\frac{1}{k}\sum_{i=1}^{k} {(x_{i}-\bar{x})}^{2}$$

$$\bar{x}=\frac{1}{k}\sum_{i=1}^{k} x_{i}$$

The first half of the formula ($\frac{x_{i}-\bar{x}}{S^{2}}$) reflects the level of region *i* versus the average level of the entire domain, and the second half ($\sum_{j=1}^{k} w_{ij}(x_{j}-\bar{x})$) reflects the level of region *i*’s surrounding areas versus the average level of the entire study area, which can be divided into four quadrants.

| $\frac{x_{i}-\bar{x}}{S^{2}}$ | $\sum_{j=1}^{k} w_{ij}(x_{j}-\bar{x})$ | meanings |
| --- | --- | --- |
| >0 | >0 | High level in region I, high level in surrounding region |
| <0 | <0 | Low level in region I, low level in surrounding region |
| <0 | >0 | Low level in region I, high level in surrounding region |
| >0 | <0 | High level in region I, low level in surrounding region |


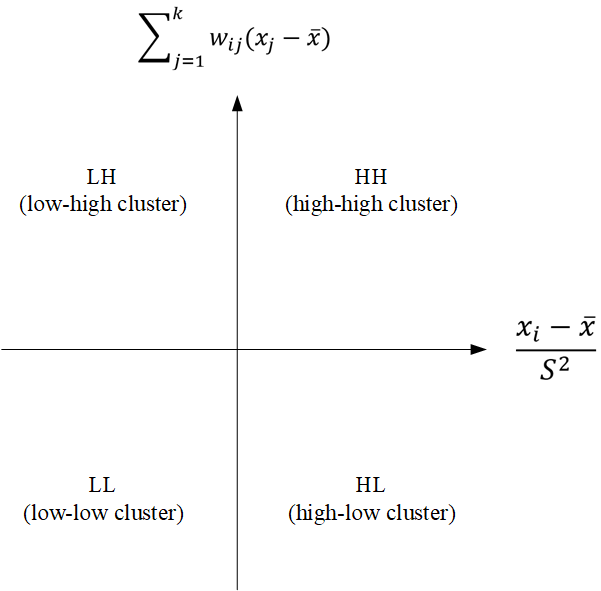


H denotes that the variable value is “greater” than the average value of the whole research region, while L denotes the “lower”. The first quadrant (H-H) indicates that high-value areas are surrounded by high-value neighborhoods, the second quadrant (H-L) indicates that high-value areas are surrounded by low-value neighborhoods, the third quadrant (L-L) indicates that low-value areas are surrounded by low-value neighborhoods, and the fourth quadrant (L-H) indicates that low-value areas are surrounded by high-value neighborhoods. H-H and L-L show minimal differences between the area and its surrounding regions, i.e., the areas with higher or lower values are concentrated, while L-H and H-H indicate a considerable degree of difference in terms of variable values between the region and its surrounding regions [12].

The LISA diagram is presented in the form of map for those regions with statistical significance, under the given significance level (generally 0.05)

# Multi-scale geographically weighted regression (MGWR)

The MGWR model is derived from the geographically weighted regression (GWR) model and is an improvement on it. It converts the classic GWR into a general additive model (GAM) and then employs this framework to the MGWR model to calculate the standard error of local parameter estimate. It enables the calculation of bandwidth and smoothing coefficients for each covariate, which is essential when fitting traditional global models and adjusting multiple hypothesis tests [12]. The calculation formula is as follows:

$$yi=\sum_{j=1}^{k} \beta_{bwj}\left( u_{i},v_{i} \right)x_{ij}+\varepsilon_{i}$$

where $\left( u_{i},v_{i} \right)$ represents “the spatial location of the i-th country”, $yi$ represents “the response variable”, $x_{ij}$ represents “the jth explanatory variable", $\beta_{bwj}\left( u_{i},v_{i} \right)$ represents “the jth coefficient”, *k* represents “the number of explanatory variable”, and $\varepsilon_{i}$ represents “the random error term”. The bandwidth utilised by the regression coefficient of the j-th variable is denoted by *bwj* in $\beta_{bwj}$. In contrast to the GWR model, the bandwidth of the MGWR model is not fixed [13].

Any time that GWR is applied to analyze data with multiple different spatial scales, one or more scales would be incorrectly specified, which could lead to bias in parameter estimates. A more reasonable assumption is that different processes can operate at distinct spatial scales. Therefore, by recasting GWR as a generalized additive model, MGWR allows each variable to be linked with a separate bandwidth such that [14]:

$$y=\sum_{j=1}^{k} f_{j}+\varepsilon(f_{j}=\beta_{bwj}x_{j})$$

Where $f_{j}$ is smoothing function applied to the j-th explanatory variable defined by different bandwidth parameter.

*Standardizing the Variables*

Standardizing is necessary to compare the bandwidths derived from an MGWR model. The dependent and independent variables are centered at zero and have the same range of variation.

*Bandwidth Selection and Model Calibration*

In practice, MGWR is usually considered as a generalized additive model, and therefore the model can be calibrated using a back-fitting algorithm with traditional GWR as the initial estimate. The basic idea of back-fitting is to calibrate each term in the model using a smoother assuming that all the other terms are known. In terms of the MGWR model, the smoother is the GWR estimator. This involves consecutively calibrating a succession of univariate GWR models depending on the partial residuals from the preceding iteration until the MGWR model converges to a solution. The flowchart of the back-fitting algorithm for calibrating the MGWR model could be found in Figure 1 of literature 15 [15]. First, all the additive terms need to be initialized, which means that all the local coefficients need to be given initial estimates. Using these initial values, an initial set of estimates of *y* is obtained, as well as a set of residuals.

$$\varepsilon=y-\sum_{j=1}^{k} f_{j}$$

Where the residual ε plus the first additive term f_1_ and the first independent variable x_1_ perform traditional GWR model and find the optimal bandwidth bwj and a new column of parameter estimates f_1_ and ε to replace the previous estimates. Next, add the second additive term f_2_ to the residual, use the same regression algorithm as the second variable x_2_, and update the parameter estimates f_2_ and ε of the second variable. According to this approach, the calculation is repeated until the parameter estimates of the last independent variable X_k_ (the k-th independent variable) converges. The SOC-f (10^-5^) is the default convergence criterion, by which the process is deemed to have converged [16].

*Kernel Functions*

The kernel function is applied to the distance between the observation and calibration points to calculate the weight matrix. The adaptive bi-square kernel is the default choice for MGWR program, and has an straightforward interpretation: the bandwidth parameter is the spatial distance or number of nearest neighbors that the remaining observations have no influence [14].

*Model Fit*

R^2^ and AICc were generally adopted to assess model fit for MGWR, better accounting for model complexity [17].

# Socio-Demographic Index (SDI) Definitions and Method

The SDI is a composite indicator of development status strongly correlated with health outcomes, ranging from 0 to 1. In short, it is the geometric mean of 0 to 1 indices of total fertility rate in those under 25 years old (TFU25), mean education for those age 15 years or older (EDU15+), and lag-distributed income per capita (LDI). A place with an SDI of 0 would have a minimal level of development relevant to these health outcomes, while a location with an SDI of 1 would have a maximum degree of development relevant to these health outcomes [18; 19; 20].

Table 1. Final scales for socio-demographic indicators for GBD 2019

| Input | Lower Bound | Upper Bound |
| --- | --- | --- |
| TFU25 | 0 | 3 |
| LDI per capita | 250 USD (5.52 log USD) | 60,000 USD (11.00 log USD) |
| EDU15+ | 0 years | 17 years |

Using scales described above, we computed the index scores underlying SDI as follows:

**$I_{CLY}=\frac{C_{LY}-C_{\text{low}}}{C_{\text{high}}-C_{\text{low}}}$

Where *I_Cly_* is the index for covariate *C*, location *l*, and year *y*. The composite SDI is the geometric mean of these three indices for a given location-year. For note, the index value for TFU25 was computed as 1-*I_TFU25ly_* because lower TFU25 correspond to higher levels of development and thus higher index scores.

The SDI values by location in 2019 are provided in table below

| **Location Name** | **1990 SDI Value** | **2019 SDI Value** |
| --- | --- | --- |
| Armenia | 0.536 | 0.689 |
| Azerbaijan | 0.576 | 0.683 |
| Georgia | 0.654 | 0.702 |
| Kazakhstan | 0.602 | 0.723 |
| Kyrgyzstan | 0.532 | 0.596 |
| Mongolia | 0.465 | 0.606 |
| Tajikistan | 0.468 | 0.539 |
| Turkmenistan | 0.548 | 0.670 |
| Uzbekistan | 0.490 | 0.631 |
| Albania | 0.540 | 0.681 |
| Bosnia and Herzegovina | 0.533 | 0.718 |
| Bulgaria | 0.631 | 0.764 |
| Croatia | 0.680 | 0.794 |
| Czechia | 0.688 | 0.828 |
| Hungary | 0.659 | 0.791 |
| Montenegro | 0.701 | 0.791 |
| North Macedonia | 0.618 | 0.744 |
| Poland | 0.632 | 0.802 |
| Romania | 0.625 | 0.760 |
| Serbia | 0.626 | 0.767 |
| Slovakia | 0.656 | 0.812 |
| Slovenia | 0.726 | 0.840 |
| Belarus | 0.591 | 0.745 |
| Estonia | 0.665 | 0.835 |
| Latvia | 0.675 | 0.820 |
| Colombia | 0.670 | 0.843 |
| Republic of Moldova | 0.585 | 0.696 |
| Russian Federation | 0.695 | 0.805 |
| Ukraine | 0.653 | 0.736 |
| Australia | 0.738 | 0.839 |
| New Zealand | 0.757 | 0.840 |
| Brunei Darussalam | 0.676 | 0.823 |
| Japan | 0.791 | 0.870 |
| Republic of Korea | 0.686 | 0.878 |
| Singapore | 0.688 | 0.861 |
| Canada | 0.790 | 0.873 |
| Greenland | 0.655 | 0.761 |
| United States of America | 0.768 | 0.859 |
| Georgia | 0.742 | 0.841 |
| Argentina | 0.581 | 0.708 |
| Chile | 0.592 | 0.759 |
| Uruguay | 0.581 | 0.697 |
| Andorra | 0.834 | 0.894 |
| Austria | 0.753 | 0.849 |
| Belgium | 0.746 | 0.851 |
| Cyprus | 0.662 | 0.841 |
| Denmark | 0.806 | 0.890 |
| Finland | 0.757 | 0.856 |
| France | 0.738 | 0.834 |
| Germany | 0.819 | 0.898 |
| Greece | 0.682 | 0.794 |
| Iceland | 0.764 | 0.869 |
| Ireland | 0.730 | 0.867 |
| Israel | 0.717 | 0.803 |
| Italy | 0.712 | 0.801 |
| Luxembourg | 0.815 | 0.895 |
| Malta | 0.666 | 0.801 |
| Monaco | 0.834 | 0.902 |
| Netherlands | 0.796 | 0.883 |
| Norway | 0.807 | 0.913 |
| Portugal | 0.607 | 0.743 |
| San Marino | 0.814 | 0.884 |
| Spain | 0.647 | 0.767 |
| Sweden | 0.769 | 0.872 |
| Switzerland | 0.868 | 0.929 |
| United Kingdom | 0.745 | 0.847 |
| Bolivia (Plurinational State of) | 0.412 | 0.566 |
| Ecuador | 0.503 | 0.640 |
| Peru | 0.501 | 0.648 |
| Antigua and Barbuda | 0.579 | 0.743 |
| Bahamas | 0.692 | 0.796 |
| Barbados | 0.649 | 0.742 |
| Belize | 0.428 | 0.603 |
| Bermuda | 0.685 | 0.813 |
| Cuba | 0.578 | 0.668 |
| Dominica | 0.579 | 0.729 |
| Dominican Republic | 0.425 | 0.592 |
| Grenada | 0.463 | 0.669 |
| Guyana | 0.452 | 0.618 |
| Haiti | 0.307 | 0.432 |
| Jamaica | 0.542 | 0.684 |
| Puerto Rico | 0.670 | 0.814 |
| Saint Kitts and Nevis | 0.583 | 0.746 |
| Saint Lucia | 0.483 | 0.670 |
| Saint Vincent and the Grenadines | 0.462 | 0.627 |
| Suriname | 0.498 | 0.636 |
| Trinidad and Tobago | 0.618 | 0.757 |
| United States Virgin Islands | 0.667 | 0.799 |
| Colombia | 0.478 | 0.633 |
| Costa Rica | 0.532 | 0.680 |
| El Salvador | 0.390 | 0.573 |
| Guatemala | 0.315 | 0.526 |
| Honduras | 0.330 | 0.496 |
| Mexico | 0.507 | 0.649 |
| Nicaragua | 0.338 | 0.517 |
| Panama | 0.544 | 0.686 |
| Venezuela (Bolivarian Republic of) | 0.509 | 0.607 |
| Brazil | 0.487 | 0.640 |
| Paraguay | 0.465 | 0.638 |
| Afghanistan | 0.187 | 0.343 |
| Algeria | 0.436 | 0.652 |
| Bahrain | 0.553 | 0.751 |
| Egypt | 0.403 | 0.658 |
| Iran (Islamic Republic of) | 0.404 | 0.670 |
| Iraq | 0.392 | 0.671 |
| Jordan | 0.520 | 0.731 |
| Kuwait | 0.655 | 0.851 |
| Lebanon | 0.462 | 0.708 |
| Libya | 0.405 | 0.709 |
| Morocco | 0.347 | 0.548 |
| Oman | 0.441 | 0.783 |
| Palestine | 0.314 | 0.588 |
| Qatar | 0.585 | 0.830 |
| Saudi Arabia | 0.480 | 0.805 |
| Sudan | 0.227 | 0.515 |
| Syrian Arab Republic | 0.367 | 0.619 |
| Tunisia | 0.434 | 0.672 |
| Turkey | 0.473 | 0.748 |
| United Arab Emirates | 0.621 | 0.880 |
| Yemen | 0.176 | 0.412 |
| Bangladesh | 0.267 | 0.483 |
| Bhutan | 0.228 | 0.455 |
| India | 0.327 | 0.566 |
| Nepal | 0.198 | 0.422 |
| Pakistan | 0.247 | 0.449 |
| China | 0.433 | 0.686 |
| Democratic People's Republic of Korea | 0.431 | 0.558 |
| Taiwan (province of China) | 0.667 | 0.868 |
| American Samoa | 0.606 | 0.712 |
| Cook Islands | 0.625 | 0.764 |
| Fiji | 0.527 | 0.664 |
| Guam | 0.693 | 0.813 |
| Kiribati | 0.425 | 0.527 |
| Marshall Islands | 0.398 | 0.544 |
| Micronesia (Federated States of) | 0.447 | 0.580 |
| Nauru | 0.499 | 0.618 |
| Niue | 0.566 | 0.711 |
| Northern Mariana Islands | 0.692 | 0.771 |
| Palau | 0.621 | 0.738 |
| Papua New Guinea | 0.292 | 0.394 |
| Samoa | 0.531 | 0.641 |
| Solomon Islands | 0.279 | 0.407 |
| Tokelau | 0.427 | 0.626 |
| Tonga | 0.510 | 0.636 |
| Tuvalu | 0.426 | 0.589 |
| Vanuatu | 0.361 | 0.485 |
| Cambodia | 0.266 | 0.469 |
| Indonesia | 0.452 | 0.660 |
| Lao People's Democratic Republic | 0.268 | 0.490 |
| Malaysia | 0.542 | 0.737 |
| Maldives | 0.303 | 0.562 |
| Mauritius | 0.527 | 0.705 |
| Myanmar | 0.284 | 0.521 |
| Philippines | 0.497 | 0.623 |
| Seychelles | 0.567 | 0.724 |
| Sri Lanka | 0.504 | 0.690 |
| Thailand | 0.508 | 0.687 |
| Timor-Leste | 0.274 | 0.514 |
| Angola | 0.238 | 0.470 |
| Central African Republic | 0.186 | 0.274 |
| Congo | 0.364 | 0.568 |
| Democratic Republic of the Congo | 0.260 | 0.382 |
| Equatorial Guinea | 0.208 | 0.685 |
| Gabon | 0.388 | 0.656 |
| Burundi | 0.198 | 0.284 |
| Comoros | 0.274 | 0.455 |
| Djibouti | 0.275 | 0.459 |
| Eritrea | 0.198 | 0.396 |
| Ethiopia | 0.144 | 0.343 |
| Kenya | 0.333 | 0.508 |
| Madagascar | 0.265 | 0.396 |
| Malawi | 0.213 | 0.384 |
| Mozambique | 0.120 | 0.307 |
| Rwanda | 0.257 | 0.429 |
| Somalia | 0.508 | 0.810 |
| South Sudan | 0.248 | 0.363 |
| Uganda | 0.167 | 0.404 |
| United Republic of Tanzania | 0.260 | 0.423 |
| Zambia | 0.299 | 0.505 |
| Botswana | 0.431 | 0.634 |
| eSwatini | 0.392 | 0.577 |
| Lesotho | 0.321 | 0.507 |
| Namibia | 0.454 | 0.612 |
| South Africa | 0.552 | 0.678 |
| Zimbabwe | 0.394 | 0.476 |
| Benin | 0.209 | 0.352 |
| Burkina Faso | 0.125 | 0.257 |
| Cabo Verde | 0.292 | 0.525 |
| Cameroon | 0.313 | 0.490 |
| Chad | 0.108 | 0.238 |
| Côte d'Ivoire | 0.256 | 0.408 |
| Gambia | 0.218 | 0.399 |
| Ghana | 0.355 | 0.557 |
| Guinea | 0.175 | 0.325 |
| Guinea-Bissau | 0.200 | 0.355 |
| Liberia | 0.221 | 0.370 |
| Mali | 0.126 | 0.263 |
| Mauritania | 0.308 | 0.496 |
| Niger | 0.728 | 0.162 |
| Nigeria | 0.305 | 0.515 |
| Sao Tome and Principe | 0.299 | 0.502 |
| Senegal | 0.227 | 0.389 |
| Sierra Leone | 0.207 | 0.347 |
| Togo | 0.266 | 0.417 |

# References

[1] H.J. Kim, M.P. Fay, E.J. Feuer, and D.N. Midthune, Permutation tests for joinpoint regression with applications to cancer rates. Stat Med 19 (2000) 335-51.

[2] Joinpoint — Joinpoint Help System [<https://surveillance.cancer.gov/help/joinpoint/>] accessed 2022-04-29.

[3] F. Wang, Y. Yu, S. Mubarik, Y. Zhang, X. Liu, Y. Cheng, et al., Global Burden of Ischemic Heart Disease and Attributable Risk Factors, 1990-2017: A Secondary Analysis Based on the Global Burden of Disease Study 2017. Clin Epidemiol 13 (2021) 859-870.

[4] M. Seeger, Gaussian processes for machine learning. Int J Neural Syst 14 (2004) 69-106.

[5] P. Mehdipour, I. Navidi, M. Parsaeian, Y. Mohammadi, M. Moradi Lakeh, E. Rezaei Darzi, et al., Application of Gaussian Process Regression (GPR) in estimating under-five mortality levels and trends in Iran 1990 - 2013, study protocol. Arch Iran Med 17 (2014) 189-92.

[6] Gaussian processes [<http://krasserm.github.io/2018/03/19/gaussian-processes/#References>] accessed 2022-04-29.

[7] Gaussian Process, not quite for dummies [<https://yugeten.github.io/posts/2019/09/GP/>] accessed 2022-04-29.

[8] G. Skolidis, and G. Sanguinetti, Bayesian multitask classification with Gaussian process priors. IEEE Trans Neural Netw 22 (2011) 2011-21.

[9] C. Liu, B. Wang, S. Liu, S. Li, K. Zhang, B. Luo, et al., Type 2 diabetes attributable to PM2.5: A global burden study from 1990 to 2019. Environ Int 156 (2021) 106725.

[10] E. Zangiacomi Martinez, and D.L. da Roza, Ecological analysis of adolescent birth rates in Brazil: Association with Human Development Index. Women Birth 33 (2020) e191-e198.

[11] L. Anselin, Local Indicators of Spatial Association—LISA. Geographical Analysis 27 (1995) 93-115.

[12] S. Tan, M. Zhang, A. Wang, X. Zhang, and T. Chen, How do varying socio-economic driving forces affect China's carbon emissions? New evidence from a multiscale geographically weighted regression model. Environ Sci Pollut Res Int 28 (2021) 41242-41254.

[13] D.X. Tran, D. Pearson, A. Palmer, J. Lowry, D. Gray, and E.J. Dominati, Quantifying spatial non-stationarity in the relationship between landscape structure and the provision of ecosystem services: An example in the New Zealand hill country. Sci Total Environ 808 (2022) 152126.

[14] T.M. Oshan, Z. Li, W. Kang, L.J. Wolf, and A.S. Fotheringham, MGWR: A Python Implementation of Multiscale Geographically Weighted Regression for Investigating Process Spatial Heterogeneity and Scale. International Journal of Geo-Information 8 (2019) 269.

[15] S.o.G. Sciences, U. Planning, A.S. University, S. Geosciences, and U. Andrews, Multiscale Geographically Weighted Regression (MGWR). Annals of the American Association of Geographers (2017).

[16] S. Tan, M. Zhang, A. Wang, X. Zhang, and T. Chen, How do varying socio-economic driving forces affect China's carbon emissions? New evidence from a multiscale geographically weighted regression model. Environmental Science and Pollution Research (2021).

[17] P. Harris, A.S. Fotheringham, R. Crespo, and M. Charlton, Inference in multiscale geographically weighted regression. WILEY 43 (2018) 399-399.

[18] G.B.D. Diseases, and C. Injuries, Global burden of 369 diseases and injuries in 204 countries and territories, 1990-2019: a systematic analysis for the Global Burden of Disease Study 2019. Lancet 396 (2020) 1204-1222.

[19] G.B.D. Disease, I. Injury, and C. Prevalence, Global, regional, and national incidence, prevalence, and years lived with disability for 310 diseases and injuries, 1990-2015: a systematic analysis for the Global Burden of Disease Study 2015. Lancet 388 (2016) 1545-1602.

[20] G.B.D. Disease, I. Injury, and C. Prevalence, Global, regional, and national incidence, prevalence, and years lived with disability for 354 diseases and injuries for 195 countries and territories, 1990-2017: a systematic analysis for the Global Burden of Disease Study 2017. Lancet 392 (2018) 1789-1858.
